# The changes of microbial diversity and flavor compounds during the fermentation of millet Huangjiu, a traditional Chinese beverage

**DOI:** 10.1371/journal.pone.0262353

**Published:** 2022-01-05

**Authors:** Yi Yan, Haiyan Chen, Leping Sun, Wei Zhang, Xin Lu, Zhenpeng Li, Jialiang Xu, Qing Ren

**Affiliations:** 1 School of Light Industry, Beijing Technology and Business University, Beijing, China; 2 Key Laboratory of Brewing Molecular Engineering of China Light Industry, Beijing, China; 3 College of Food Science and Technology, Hebei Agricultural University, Baoding, China; 4 State Key Laboratory for Infectious Disease Prevention and Control, National Institute for Communicable Disease Control and Prevention, Chinese Center for Disease Control and Prevention, Beijing, China; SRUC: Scotland’s Rural College, UNITED KINGDOM

## Abstract

Huangjiu is a national alcoholic beverage in China. Millet has congenital advantages in development and utilization of nutrient. Brewing Huangjiu with millet can increase the value of millet. Microbial community plays crucial roles in millet Huangjiu fermentation. Flavor compounds reflect the quality and health function of Huangjiu. The flavor compounds of Huangjiu are complex and their formation is closely associated with microorganisms, but the relationship between them during fermentation has been unknown. In this research, this relationship during millet Huangjiu fermentation were deeply investigated. Totally 86 volatile compounds were detected. *Bacillus*, *Weissella*, *Paenibacillus*, *Klebsiella*, *Prevotella* was investigated as the dominant microbes through high-throughput sequencing. 537 correlations between major flavor compounds and microbes were established to reflect the dynamic change during millet Huangjiu fermentation. The top five dominant genus of flavor producing microbes were *Chryseobacterium*, *Sporolactobacillus*, *Psychrobacter*, *Sphingobium* and *Anoxybacillus*. The content of malic acid and citric acid was gradually improved all through the millet Huangjiu fermentation. Malic acid and citric acid generated from millet Huangjiu fermentation shows healthy properties as liver protection and eliminating fatigue. Our research provides essential information on microbial community succession and the flavor formation during millet Huangjiu fermentation, and beneficial for development of Huangjiu products.

## Introduction

Huangjiu is one of the oldest wine types and most famous liquors in China. Over the past several years, the joint efforts of the entire industry promoted the rapid development of rice wine, which also attract notable attention [1]. Glutinous rice was usually used as the brewing raw materials, such as Guyue Longshan rice wine in southern China. Non-glutinous rice, indica rice and glutinous rice mentioned earlier are three typical raw materials for traditional Huangjiu brewing, barleys and millet with prominent regional characteristics also beginning to emerge [2, 3]. The key raw material in Northern Huangjiu is millet, the content of crude protein and starch in which is much higher than that of rice and wheat [4, 5]. As excellent natural functional food with high nutritious value, millet shows unique advantages in food development and application, which has been validated in yogurt, bread and baby supplementary foods [6, 7]. All these superior qualities make it very suitable for Northern Huangjiu fermentation. Furthermore, dietary fiber, flavonoids, polyphenols, inositol, sterols and other nutrients are also found rich in millet meanwhile [8, 9]. On the one hand, current studies predominantly focus on the improvement of millet brewing technology, the flavor formation in which was comparatively less well understood [10–12]. On the other hand, the exploitation and utilization of millet resources has been limited due to its indigestion [13]. Huangjiu brewed by millet could increase the value of millet, which also have some implications for the development of Huangjiu products.

The aroma is the best characterized feature of Huangjiu, determined by diverse volatile flavor compounds, and over 900 different kinds of various volatile flavor compounds have been confirmed according Chen et al’s study, comprising primarily esters, alcohols, phenols, aldehydes, ketones and acids [14, 15].Though the classification of Huangjiu varies according to region or aroma types, the production process could be generally divided into six major stages [3, 15–17]: the pretreatment of raw materials (especially soaking in millet Northern Huangjiu, while glutinous rice steaming in Southern Huangjiu), sacccharification (primary fermentation), alcoholization (secondary fermentation), filtering, sterilizing and aging. In other words, almost all the aroma compounds have been achieved through the above complicated fermentation process with the help of raw materials and Qu (fermentation starter) [18], during which the microbial diversity plays the most critical and indispensable role [19, 20]. Microbiota and flavor dynamics during Huangjiu brewing mainly refers to simultaneous saccharification fermentation [3, 21]. Several studies have demonstrated that *Bacillus*, *Leuconostoc*, *Lactococcus*, *Weissella*, *Thermoactinomyces*, *Pseudomonas*, *Saccharopolyspora*, *Staphylococcus*, *Enterobacter* and *Lactobacillus* were the dominant genera during the fermentation of Shaoxing rice wine [21]. Analogously, five *Bacillus* species and three lactic acid bacteria were identified as the dominant bacteria in Hong Qu rice wine, which is another eminent Chinese Huangjiu as well [22]. These microorganisms have been proved essential during fermentation and the generation of special flavors. Chen found that the main fungi species during wheat Qu storage were *Aspergillus oryzae*, *Absidia corymbifera*, *Rhizomucor pusillus*, *Clavispora lusitaniae* and *Saccharomycopsis fibuligera* [23]. Another study combined traditional microorganism isolation methods and PCR- Denaturing Gradient Gel Electrophoresis (DGGE) technology obtained *Thermomyces lanuginosus* and *Fusarium sp*., which had not been reported in Shaoxing Huangjiu wheat Qu [24]. According to Huang et al, *Lactobacillus*, *Leuconostoc*, and *Bacillus* from bacteria, and *Weissella*, *Saccharomyces*, *Rhizopus*, *Aspergillus* and *Candida* of fungi are the core functional microorganisms during Wuyi Hongqu Huangjiu fermentation [16].It has been reported that six microbial genera (*Saccharomyces*, *Aspergillus*, *Saccharopolyspora*, *Staphylococcus*, *Lactobacillus*, and *Lactococcus*) were most intimately linked to the major flavor components-amino acids, alcohols, acids, phenols and esters [25].

This study aims at monitoring the bacterial succession via high throughput sequencing (HTS) and the detection of the volatile compound dynamics with the help of headspace solid phase micro-extraction combined with gas chromatography-mass spectrometry (HS-SPME/GC-MS) during brewing. We also considered to find the relationship between volatile compounds and the bacterial flora, expecting to provide promising perspectives on flavor and functional microbes in millet Huangjiu fermentation for the first time. Usage of millet brewed Huangjiu can realize the development and utilization of millet resources, and is beneficial for the development of novel Huangjiu products.

## Materials and methods

### Sample collection

The fermentation assay was displayed at a constant temperate of 28°C and the fermentation stage lasted for twelve days. The fermented mash of each sample was collected at 0, 2, 4, 6, 8, 10, 12 fermentation stages. Each sample has six replications. Each sample was conducted for flavor test and high-throughput sequencing.

### Determination of reducing sugar, alcohol, acidity, volatile compounds and organic acids

The dinitrosalicylic acid method (DNS) was used to detect the content of reducing-sugar with glucose as standard substance [26]. The alcohol degree and the acidity assay could evaluate the quality of Huangjiu, which was measured based on the standard of GB/T13662-2008.

The HS-SPME-GC-MS was used to analyze the characters of volatile compounds. The volatile compounds were collected by a 50/30μm DVB/CAR/PDMS (Superco, Bellefonte, PA, USA). Each Huangjiu sample (8 mL) was set in a 15 mL SPME glass vial in addition with 2.5 g NaCl and 5 mL internal standards (65.76 mg/L 2-octanol). Then the water bath and ultrasonic wave were applied for the treatment of the mixture for 45 min at 50°C. The volatile compounds were identified via a Shimadzua-QP2010 Plus-GCMS. The carrier gas helium was circulated at the speed of 1 mL/min with the split-flow mode, the split ratio of which was set as 50/1. The settings of the oven temperature program were as follows: 35°C 4 min; five centigrade per minute ramp to 150°C and time-keeping for 2 min; 3°C/min ramp to 210°C. The temperatures of detector and injector were both 230°C and that of ion source was 200°C. The ion energy for electron impact (EI) was adjusted to 70 eV. The detection and monitor of the total ion currents were performed to record the chromatograms in 30–350 mass range. 2-octanol was used as the internal standard to determine semi-quantitatively the content of the volatile compounds [27].

The HPLC analysis was used to analyze organic acids. Each wine sample of 5 mL was put in tube and centrifuged at 10,000 r/min for 20 min, then filtrated through a microporous membrane, which was 0.45 mm. Chromatographic conditions were referred to the method proposed by Ye et al with some modifications [28]. The separations were conducted on Agilent 1260 Infinity II equipped with a 250 mm x 4.6 mm and 5 μm welch ultimate XB-C18 column. The temperature of the column was set at 30°C. A mixture of phosphate buffer (0.01 mol/L (NH_4_)_2_HPO_4_), adjusted with the solution of phosphoric acid to pH 3.0 was employed as the mobile phase with a flow rate of 0.7 mL/min. The detection wavelength was 215 nm.

### DNA extraction, and high-throughput sequencing

Total genomic DNA of fermented mash samples was extracted via CTAB method, which was further adjusted to a uniform final concentration of 1 ng/μL by sterile water.

Hypervariable regions of V3-V4 on 16S rRNA gene of bacteria were amplified through PCR with specific primers of 338 F (5′-ACTCCTACGGGAGGCAGCAG-3′) and 806 R (5′-GGACTACHVGGGTWTCTAAT-3′). Primers of ITS1 (5′-AxxxCTTGGTCATTTAGAGGAAGTAA-3′) and ITS2 (5′-BGCTGCGTTCTTCATCGATGC-3′) were used to amplified ITS1 and ITS2 region of fungus. All PCR reactions were performed in Phusion High-Fidelity PCR Master Mix (NEB). The mixture of PCR products was then purified using Qiagen Gel Extraction Kit (Qiagen, Germany). Sequencing libraries were generated using TruSeq® DNA PCR-Free Sample Preparation Kit (Illumina, USA) following the manufacturer’s recommendations. The library quality was assessed on the Agilent Bioanalyzer 2100 system. Finally, the library mentioned above was sequenced with the help of an Illumina HiSeq2500 platform.

Paired-end reads were assigned to samples on account of their unique barcode and truncated through cutting off the primer sequences and barcode. Paired-end reads were merged via FLASH (V1.2.7) [29]. According to the QIIME (V1.7.0), the high-quality clean reads were obtained after the raw reads filtered under specific filtering conditions [30, 31]. Chimera sequences were detected through the reference database (Gold database) using UCHIME algorithm, and then removed out [32, 33].

### OTU cluster, species annotation and phylogenetic analysis

Sequences were performed with Uparse software (Uparse v7.0.1001) [34]. Sequences with ≥97% similarities were assigned to be the same OTUs. GreenGene Database based on RDP 3 classifier (Version 2.2) was applied to annotate the taxonomic information for each representative OTU [35, 36]. Alpha and beta diversity analysis was performed on account of the normalized data. The phylogenetic relationship of different OTUs was investigated by multiple sequence alignment by the MUSCLE software (Version 3.8.31) [37]. Alpha diversity was analyzed via the usage of six indices, including Observed-species, Chao1, Shannon, Simpson, ACE and coverage. All the indices were calculated with QIIME (Version 1.7.0) and displayed with R software (Version 2.15.3). Beta diversity was calculated by both weighted and unweighted unifrac via QIIME software (Version 1.7.0).

### Correlations between microorganisms and flavor compounds

Correlations between the microorganisms and flavor compounds during the fermentation of millet Huangjiu were established by Pearson correlation coefficient (r). R programming language was used to construct the correlation network. The P value was adjusted by FDR using the Benjamini-Hochberg method. P value and the adjusted P value lower than 0.05 was regarded as significant difference.

## Results and discussion

### The acid, reducing sugar and alcohol were altered during millet Huangjiu fermentation

**Fig 1:**
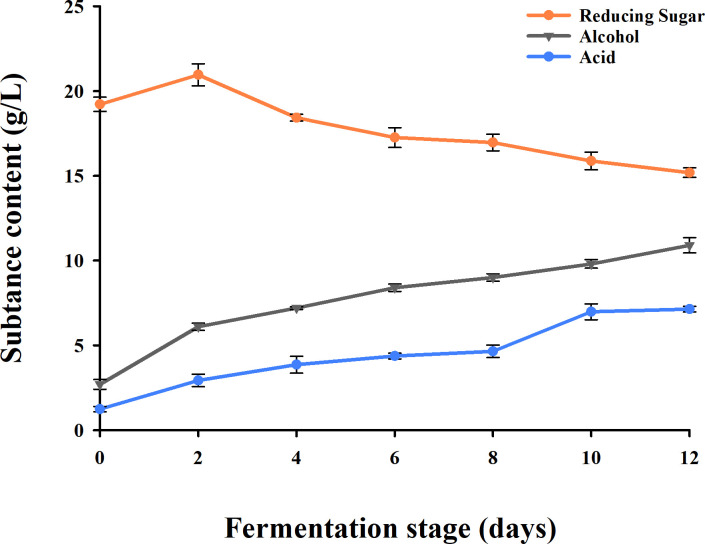
Dynamic change of the content of acid, reducing sugar and alcohol in millet Huangjiu fermentation during different stages. X-axis means fermentation stages, Y-axis represents substance contents.

The contents of acids, reducing sugar and alcohol were detected based on National Standard of the People’s Republic of China [38]. The results showed acid concentration continued to climb with a rapid growth since 8th day then began to flatten at day 10. The content of reducing sugar reached peak value on the 2nd day, and decreased progressively with the process of fermentation. The alcohol also tended to increase gradually and exhibited a fast increase from day 0 to day 2, with a steady growth rate from 4th day to 12th day (Fig 1).

Studies on Huangjiu using traditional rice as raw material have shown that the liquor output rate was negatively correlated to the fat content in rice. In the meantime, the reduction of protein in raw materials could effectively control the peculiar smell raised by excessive higher alcohols, and the contents of harmful substances such as ethyl urethane and biogenic amine in wine. Furthermore, the starch granules expanded after water absorption, facilitating the accumulation of reducing sugar, helpful for further saccharification and fermentation in millet Huangjiu. Therefore, the application of millet with low concentration of protein and fat, as well as high content of starch as raw material for Huangjiu brewing would be a better choice [39–42].

### Organic acid in millet Huangjiu fermentation

Acid is an important flavoring substance of wine. Its main component organic acid, an essential precursor of flavors, could interact with other aroma & flavor-producing substances then collectively form the unique flavor and fragrance in Huangjiu [43, 44]. An appropriate amount of organic acid helps harmonize and stabilize the taste and aroma of wine, making it refreshing and palatable. Moreover, organic acids also hold considerable implications for improving intestinal function, resisting fatigue and other health benefits [45]. The alcoholic and malolactic fermentation and oxidation of the ethanol contributed a lot to the formation of organic acids.

The main organic acids identified in millet Huangjiu fermentation were oxalic acid, tartaric acid, pyruvic acid, malic acid, α-ketoglutaric acid, lactic acid, citric acid and succinic (S1 Table). The concentration of oxalic acid, tartaric acid, malic acid, lactic acid, citric acid and succinic acid were showed to be upward in general as the fermentation time extended compared to day 0. Most of them have increased sharply from 8th day to 10th day. As a whole, the total content of organic acids reached the peak at the end of the fermentation day 12, while the valley was detected at the initial day 0. The concentration of succinic acid and lactic acid were significantly higher than others, especially the former. Pyruvic acid reached the highest concentration at 6th day, subsequently underwent a decline then increased a bit on day 10 and 12 but still lower than the value of day 6. α-ketoglutaric acid reached its peak on 2nd day, and then kept decreasing over the remnant days, becoming the least abundant substance al last.

Lactic acid, malic acid, citric acid, tartaric acid and succinic acid are typical non-volatile acid [46]. Lactic acid bacteria presented in Huangjiu fermentation could produce massive lactic acid, which contribute a lot to the excellent flavor of Huangjiu. Malic acid is widely known for anti-fatigue, liver protection and cardiovascular fitness enhancement, while citric acid mainly serves as delaying senility, lowering blood pressure and eliminating fatigue. They have shown important effects on the quality of Huangjiu.

### Volatile compounds in in millet Huangjiu fermentation

In this study, 95 volatile flavor compounds were finally detected during the whole fermentation process, including 31 esters, 23 alcohols, 13 alkanes, 7 ketones, 6 acids, 3 phenols, 2 aldehydes and 10 other kinds of volatile compounds (S2 Table). Hexadecanoic acid ethyl ester, 9,12-octadecadienoic acid ethyl ester, decanoic acid ethyl ester and hexanoic acid ethyl ester were the dominant ester with high concentration in the last day of fermentation. Among all detectable alcohols, 3-methyl-1-butanol, 2,3-butanediol, phenylethyl alcohol and glycerin were major alcohols during the fermentation process. In addition, 2-methoxy-4-vinylphenol, pentacosane and 2-octanone were the dominant phenol, alkane and ketones, respectively. At the last day of fermentation, 48 compounds were detected, including twenty-four esters, thirteen alcohols, three ketones, three acids, one alkane, one aldehyde, one phenol, and three other volatile compounds. The contents and varieties of those flavor compounds reached their peak at 10^th^ day.

The types and quantities of flavor compounds were fluctuated during the different brewing stages. The content of alcohol was relatively steady in different brewing periods; the changes of esters and alcohol were stable except for a mild decrease at the end of brewing. While the content of acids and alkanes were gradually declined with the development of brewing.

The characteristic flavor compounds in fermented millet Huangjiu are 2-methyl-1-propanol, 3-methyl-1-butanol, phenethyl alcohol, hexanoic acid ethyl ester, octanoic acid ethyl ester, decanoic acid ethyl ester, 2-hydroxy-propanoic acid ethyl ester, hexadecanoic acid ethyl ester, (E)-9-octadecenoic acid ethyl ester, 9,12-octadecadienoic acid ethyl ester.

### Bacterial community dynamics during millet Huangjiu fermentation

The initial fermentation had significant difference in bacterial richness and community diversity compared with later fermentation. Bacterial richness was not altered during the middle and final stages of fermentation, but the community diversity in the two stages were dramatically different (Table 1 and S1 Fig).

**Table 1:** Comparison of α diversity and β diversity during millet Huangjiu fermentation.

Between-group	P-values (α Diversity Index)					P-values (β Diversity Index)
	Observed_ species	Chao1	ACE	Shannon	Simpson	Weighted Unifrac distance
Day0 vs Day2	**	*	*	***	NS	NS
Day0 vs Day4	*	NS	NS	**	***	**
Day0 vs Day6	*	*	NS	**	NS	NS
Day0 vs Day8	NS	NS	NS	NS	NS	NS
Day0 vs Day10	**	**	*	*	*	**
Day0 vs Day12	*	*	*	***	***	*
Day2 vs Day4	NS	NS	NS	*	**	NS
Day2 vs Day6	NS	NS	NS	NS	*	NS
Day2 vs Day8	NS	NS	NS	**	NS	NS
Day2 vs Day10	NS	NS	NS	*	***	NS
Day2 vs Day12	NS	NS	NS	NS	*	NS
Day4 vs Day6	NS	NS	NS	NS	NS	NS
Day4 vs Day8	NS	NS	NS	NS	**	NS
Day4 vs Day10	NS	NS	NS	NS	NS	NS
Day4 vs Day12	NS	NS	NS	NS	NS	NS
Day6 vs Day8	NS	NS	NS	*	***	NS
Day6 vs Day10	NS	NS	NS	NS	**	NS
Day6 vs Day12	NS	NS	NS	NS	NS	NS
Day8 vs Day10	*	*	NS	NS	NS	NS
Day8 vs Day12	NS	NS	NS	NS	**	NS
Day10 vs Day12	NS	NS	NS	NS	*	NS

Significance: NS > 0.05,

*≤0.05,

**≤0.01,

***≦0.001

**Fig 2:**
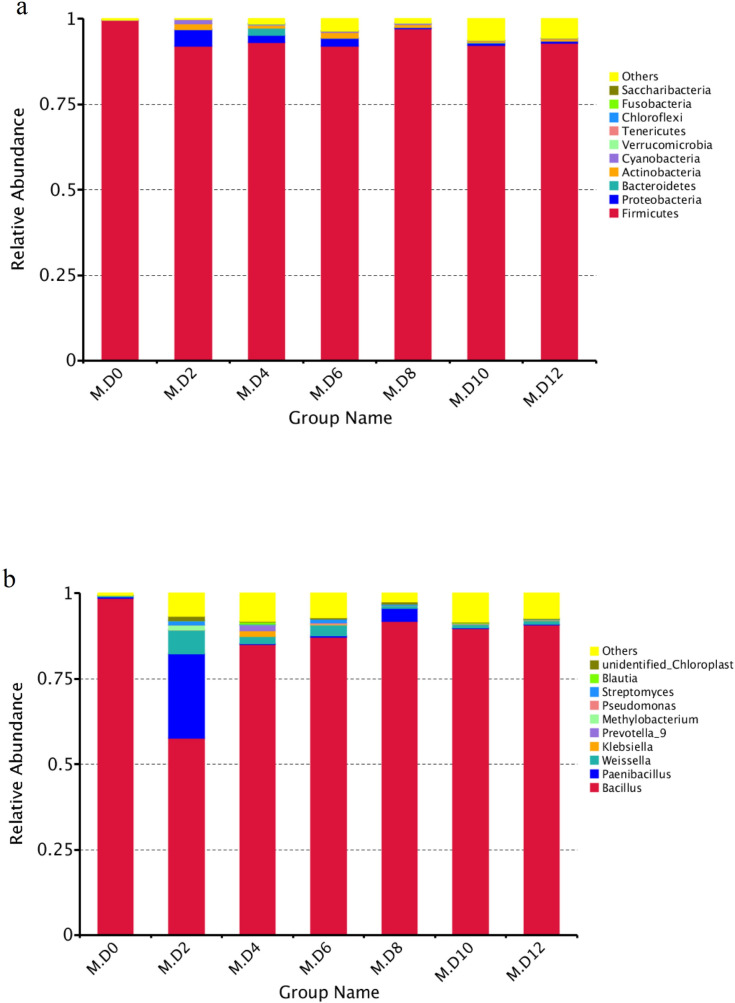
Relative abundance levels of bacterial taxon during millet Huangjiu fermentation at different stages. **(a)**: Phyla taxon; **(b)**: genus taxon.

An average of 2,455,812 high-quality reads were obtained through high-throughput sequencing. Totally, 26 phyla, 45 classes, 90 orders, 142 families and 284 genera of bacteria participated in millet Huangjiu fermentation. The dominant phyla in millet Huangjiu fermentation is *Firmicutes*, which predominated over 90% in the whole samples (Fig 2A). Followed by *Proteobacteria* and *Bacteroidetes*. *Firmicutes* was gradually declined in the first six days. *Proteobacteria* appeared its maximum quantity on the second day, and then slightly decreased. The peak of *Bacteroidetes* was appeared at the fourth day.

From the genus level (Fig 2B), *Bacillus* was predominant, represented by 57.60–98.69% among the bacteria. The second abundant genus was *Paenibacillus*, with a decline on the sixth day. *Weissella* was the third abundant genus, which reached the highest quantity on the 2^nd^ day, then decreased and gradually leveled off on the 8^th^ day.

### Fungal community dynamics during millet Huangjiu fermentation

**Fig 3:**
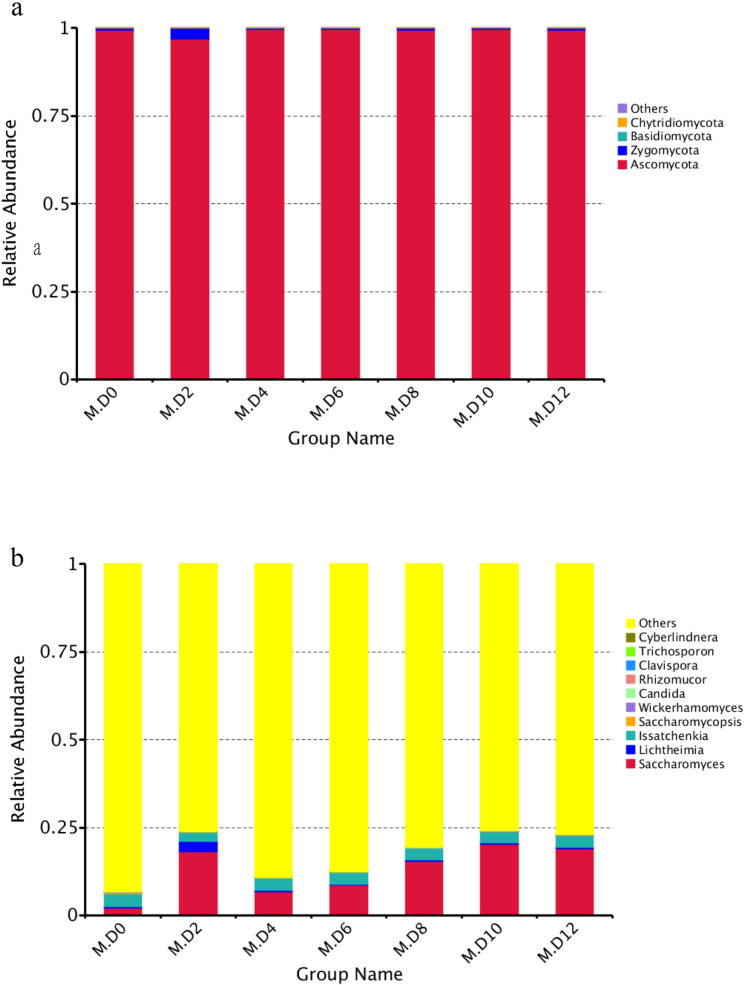
Relative abundance levels of fungal taxon during millet Huangjiu fermentation at different stages. **(a)**: Phyla taxon; **(b)**: genus taxon.

Four phyla, 9 classes, 9 orders, 13 families and 15 genera of fungus were identified and characterized. The results showed that approximately 99% fungus belong to the phylum of Ascomycota (Fig 3A). *Saccharomyces*, *Issatchenkia* and *Lichtheimia* occupied the top three at genus level (Fig 3B). *Saccharomyces* and *Lichtheimia* reached the highest level on the second day. The quantity of *Saccharomyces* was declined on the fourth day, and then gradually increased at remaining fermentation stage. The quantity of *Issatchenkia* was varied slowly in the fermentation process.

### Relationship between microorganisms and flavor compounds during millet Huangjiu fermentation

**Fig 4:**
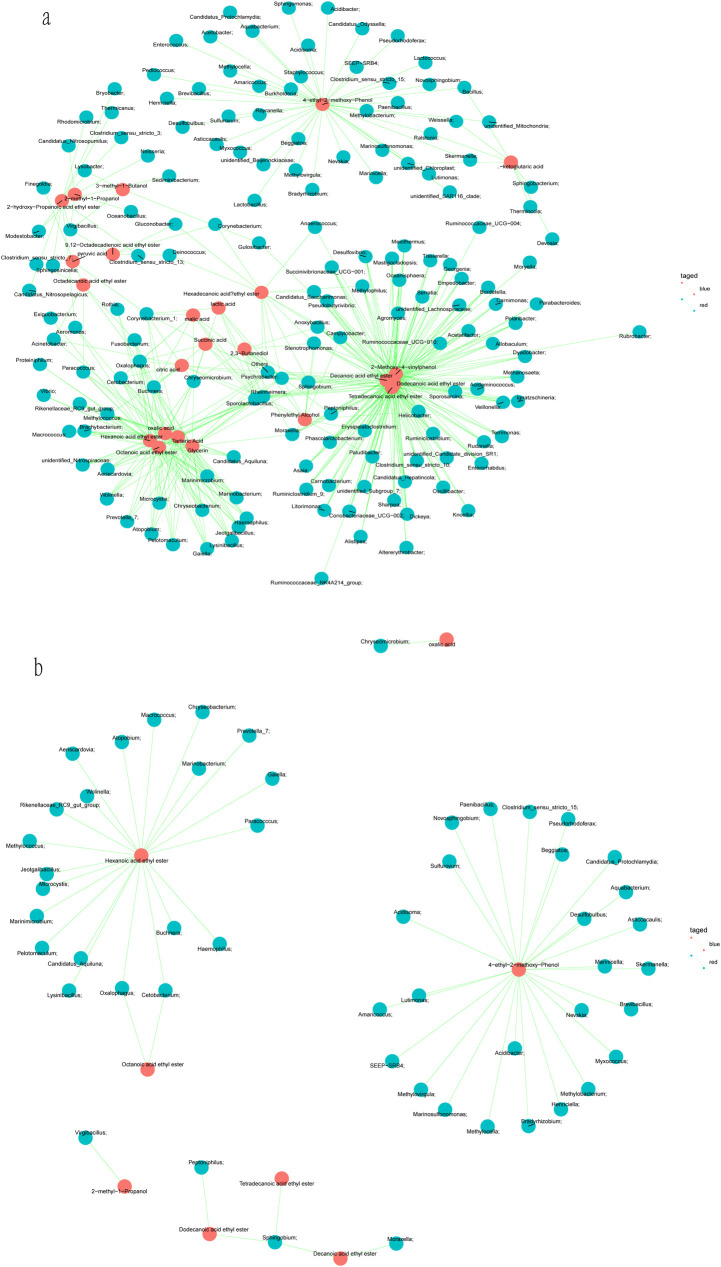
The correlation analysis between microorganisms and flavor compounds during the fermentation of millet Huangjiu. The blue pie represents microbial composition; the red pie indicates flavor compounds composition; the green line means correlation between microorganisms and flavor compounds. a: *P<0*.*05*; b: *P* adjust<0.05.

Totally 537 correlations were established between flavor compounds and microorganisms during millet Huangjiu fermentation. 153 microorganisms were relevant to the formation of main flavor compounds (*P<0*.*05*) (Fig 4A). *Rheinheimera*, *Psychrobacter*, *Sporolactobacillus*, and *Chryseomicrobium* participated in the formation of more than 10 flavor compounds. *Acetobacter*, *Asticcacaulis*, *Bradyrhizobium*, *Brevibacillus*, *Enterococcus*, *Aquabacterium*, *Methylobacterium*, *Myxococcus*, *Novosphingobium*, *and Sphingomonas* were only associated with the formation of 4-ethyl-2-methoxy-phenol. *Clostridium_sensu_stricto*_7, *Exiguobacterium*, *Finegoldia*, *Lysobacter*, was related to the generation of octadecanoic acid ethyl ester, hexanoic acid ethyl ester, octadecanoic acid ethyl ester and 3-methyl-1-butanol, respectively. There are more than 15 genera were associated with the formation of acetic acid phenylethyl ester, decanoic acid ethyl ester, 4-ethyl-2-methoxy-phenol, hexanoic acid ethyl ester, octanoic acid ethyl ester, oxalic acid, tartaric acid, glycerin and 3-hydroxy-2-butanone.

*P* value related to microorganisms and the major flavor compounds was adjusted to obtain closer correlation. 56 correlations were established by filtering *P* adjust less than 0.05 (Fig 4B). More than 20 microorganisms exhibited correlations to the formation of 4-ethyl-2-methoxy-phenol and hexanoic acid ethyl ester. *Sphingobium* showed a closer correlation with the generation of dodecanoic acid ethyl ester, tetradecanoic acid ethyl ester and decanoic acid ethyl ester. 2-methyl-1-propanol was showed a closer correlation with *Virgibacillus*. Dodecanic acid ethyl ester is colorless oil liquid with a waxy, rum aroma and cream flavors, commonly found in cognac, rum, Irish whiskey and Chinese traditional beverages [47–49], also detected in our millet Huangjiu. It could be used in daily fragrance and flavoring essence for the manufacture of soft drinks, ice creams, candies and baking food, also applied to produce lubricants, plasticizers and softeners. The traditional catalyst for synthesizing dodecanic acid ethyl ester is concentrated sulfuric acid, although cheap and easy to obtain, there have still been many side reactions [50]. The accumulation of carboid is inevitable and the color of the esterified products is too much darker. Moreover, the product quality is seriously affected and the post-reaction treatment is also complicated. Therefore, the use of biotechnology methods, such as direct microbial fermentation and enzymatic catalysis exhibit lower energy consumption and higher product quality [51]. Above all, it is more environmentally friendly than traditional chemical synthesis methods. In this study, after P value adjust (Fig 4B), we found that *Sphingobium* still has a strong correlation with the formation of dodecanic acid ethyl ester, which suggests that we could subsequently isolate and cultivate this strain and optimize the fermentation conditions to achieve the large-scale synthesis of dodecanic acid ethyl ester *in vivo*, overcoming the defects of the original production process.

Open fermentation of Huangjiu could result in the diverse microorganisms in the brewing process [52]. Microorganisms in the wheat Qu and external environment together provide essential enzymes and metabolites for the fermentation process, which has also formed the unique flavor of Huangjiu. In the present study, the main bacteria in the fermentation of millet Huangjiu were *Bacillus*, *Brevibacillus* and *Weissella*. *Bacillus* could secret carbohydrate degradation enzymes, such as glucanase, pectinase and cellulase, which could destroy the cell walls of plant cells and release the nutrients. *Bacillus* could also synthesize many different kinds of organic acids, physiological active substances and nutrients, most of which are flavor precursors and flavor compounds, which explains the dominant reason for *Bacillus* [53]. *Brevibacillus* could degrade starch, xylan, cellulose, non-starch polysaccharides and improve the utilization rate of raw materials. The function of *Weissella* in fermentation was decompose glucose to carbon dioxide and ethanol [54].

Higher alcohols and aromatic esters are the dominant and important volatile flavor components in Huangjiu. They reflect the quality and the flavor of Huangjiu. In this study, the main alcohols were 2-methyl-1-propanol, 3-methyl-1-butanol, 2-3-butanediol and β-phenethyl alcohol, most of which were the degradation products of amino acids [55]. 2-methyl-1-propanol could be obtained from natural fermentation of carbohydrates, or biosynthesized by genetic engineering techniques [56–58]. Shota Atsumi reported intermediates *Escherichia coli* amino acid synthesis pathway could generate 2-methyl-1-propanol through expressed *kivd* and *ADH2* (14). In this study, we found the formation of 2-methyl-1-propanol was closely related to *Candidatus_Nitrosopumilus*, *Modestobacter*, *Oceanobacillus* and *Virgibacillus*. 3-methyl-1-butanol could affect the quality of the Huangjiu, and is also harmful to human toxicity [59]. Our study suggested that *Gulosibacter*, *Lysobacter*, *Virgibacillus* were related to the production of 3-methyl-1-butanol. It is quite necessary to decrease the content of 3-methyl-1-butanol by regulating the three strains during Huangjiu fermentation. 2,3-butanediol has sweet taste and has been widely used to improve liquor flavor. *K*. *oxytoca* and *B*. *polymyxa* displayed a relative higher production ability of 2-3-butanediol [60, 61]. In the process of millet Huangjiu brewing, *Anoxybacillus*, *Peptoniphilus*, *Sphingobium*, *Sporolactobacillus* and *Stenotrophomonas* were closely related to the synthesis of 2,3-butanediol. Phenethyl alcohol is an aromatic higher alcohol, which is extensively used in various alcoholic beverages [27]. Previous studies showed that phenethyl alcohol was mainly produced by yeast metabolism and growth [62, 63]. In addition, *Helicobacter*, *Peptoniphilus*, *Sphingobium*, *Sporolactobacillus* and *Stenotrophomonas* were also involved in the synthesis of beta phenethyl alcohol [64].

Higher alcohols are very important precursors for ester formation. Esters can impact the quality and flowery flavors of alcohol beverages. The main esters during fermentation are identified as hexanoic acid ethyl ester (strong aroma for liquor blending), octanoic acid ethyl ester (colorless transparent liquid with an odor similar to brandy), decanoic acid ethyl ester (colorless transparent liquid with fruity and bouquet aroma) [65], hexadecanoic acid ethyl ester (light yellow oily liquid with the aroma of oil, sweet and mellow, increase the mellow feeling of the wine) [66], 9,12-octadecadienoic acid ethyl ester (responsible for reducing blood fat, preventing and curing atherosclerosis). The formation of these esters is closely related to alcohols, fatty acids, coenzyme A, etc. [67]. Their synthesis pathways are concerned with yeast species. In the present research, we found *Acidaminococcus*, *Anoxybacillus*, *Bordetella*, *Deinococcus*, *Fusobacterium*, *Gluconobacter*, *Peptoniphilus*, *Serratia* and *Sphingobium* were involved in the synthesis of various esters during millet Hunagjiu fermentation, which also indicated that the biosynthesis of esters was associated with microorganisms.

As one of the extremely important flavoring substances in Huangjiu [68] and Baijiu [69, 70], hexanoic acid ethyl ester (ethyl hexanoate) is also the representative of the characteristic flavor component in Baijiu, the content of which directly determines the quality of strong-flavor Baijiu. Esters produced during the fermentation process are mainly synthesized by the following two pathways based on present research: one is that microorganisms synthesize esters under the action of their own intracellular enzymes [71–73]; while the more frequent method is the catalysis by the extracellular enzymes (microbial lipases) secreted by microorganisms when organic acids react with alcohols in outside surrounding environment for synthesis [74, 75]. In addition, the acidic substances and alcohols in the wine body can also undergo esterification under natural conditions, but the rate and yield are far lower than the above two methods [76]. *Bacillus* spp., *Alcaligenes* spp., and *Pseudomonos* spp., of bacteria and *Penicillium* spp., *Fusarium* spp., *Aspergillus* spp., of fungi are screened as candidates for large scale of the production of lipases mentioned above [77]. However, the selection and modification of high-yield ethyl caproate strains mainly focus on *Saccharomyces cerevisiae* [78–80]and *Monascus purpureus* [81–83]. In our study, we established the closer relationship between 21 novel genus of bacterium (such as *Aeriscardovia*, *Atopobium* and *Paracocccus*) and the formation of ethyl hexanoate after P value adjust (Fig 4B). The isolation and cultivation of them would provide a solid theoretical basis for the identification of bacteria producing ethyl hexanoate esterase and the optimization of fermentation conditions for further higher yield.

With herbal aroma and warm spicy taste, 4-ethyl-2-methoxyphenol has been widely used in food additive and fragrance, especially in wine and soy sauce [84]. As Fig 4B showed, the formation of 4-ethyl-2-methoxyphenol could be related to 26 different genera of bacterium with less abundance during millet Huangjiu fermentation, suggesting that the generation of flavor compounds is sophisticated and fantastic, and it is worth noting that the interaction between different, especially those less abundant microbes.

Acid is an important flavor compounds of Huangjiu, and organic acid of which is mainly responsible for the sour taste in Huangjiu. Moreover, organic acids are critical to improve and enhance intestinal function, anti-fatigue and other health functions. Malic acid plays important roles in anti-fatigue, liver protection and cardiovascular fitness enhancement. The main function of citric acid is delaying senility, lowering blood pressure and eliminating fatigue. Most *Lactobacillus* species have been proved indispensable for producing lactic acid, ethanol and acetic acid. The genus *Saccharomyces*, *Pichia*, and *Zygosaccharomyces* could convert lactic acid to pyruvate. Moreover, there have been evidences that they could convert pyruvate to acetyl-CoA, acetaldehyde in liquor production [85]. Microorganisms include several yeast, *Aspergillus*, *Penicillium* and *Candida* could accumulate citric acid [86]. The microorganisms that could produce malic acid were *Saccharomyces* and *Aspergillus* [87, 88]. The genera *Bacteroides*, *Porphyromonas* and *Sedimentibacter* have been reported to produce succinic acid, propionic acid and alcohols [89–91]. Besides the microorganisms reported above, many other microorganisms are also involved in the formation of organic acids according to our study. *Aeromonas*, *Brachybacterium*, *Haemophilus*, *Weissella* can utilize glucose to produce acid during fermentation. *Fusobacterium* can hydrolyze sugars and proteins, and usually produce mixed organic acids and alcohols with sugar or peptone participation. Furthermore, there are numbers of microbial species such as *Buchnera*, *Cetobacterium*, *Chryseobacterium*, *Macrococcus*, *GaiellaJeotgalibacillus*, *Marinimicrobium*, *Oxalophagus*, *Wolinella*, *Pelotomaculum*, *Psychrobacter* all related to organic acid production, but have not yet been fully investigated in detail. The relationship between these microorganisms and their corresponding acids needs to be verified in future. The existence of carbonyl and carboxyl groups in pyruvate acid makes its participation in various biochemical reactions, especially in Tricarboxylic acid cycle and microbial metabolism, further promoting more microbial involvement during fermentation, also the direct or indirect precursor of many high value-added products in millet Huangjiu aroma. The principally well-known pyruvate bioproduction microorganisms are fungus like *Torulopsis glabrata* [92, 93] and *Saccharomyces cerevisiae* [94], with less research on bacterium. For example, *Corynebacterium pyruviciproducens* was isolated as candidate strains to produce pyruvate [95, 96] and *Escherichia coli aceF* mutant strains were genetically engineered to ferment pyruvate [97, 98]. In our study, *Corynebacterium* was the only genus predicted association with the formation of pyruvic acid. This strong relevance indicated it could be a high-productive pyruvate strain during millet Huangjiu fermentation, also inspired us for its further isolation and extensive application.

Millet, a kind of high-quality healthy grain, has not been paid enough attention to its utilization. Although as the main raw material for Huangjiu fermentation, it has seldom been reported. Northern Huangjiu using millet can realize the development and utilization of millet resources, and also beneficial for the development of novel Huangjiu products. On the other hand, the way to promote the quality and flavor of Huangjiu is a consistent problem. Our work could provide more suggestive information on flavor and functional microbes during millet Huangjiu fermentation. Future investigation would focus on the improvement millet Huangjiu quality by synthetic microbial communities closely relevant to aroma compounds.

## Conclusions

Millet Huangjiu is a national alcoholic beverage in China. In this study, basic physicochemical parameters, 95 flavor compounds (31 esters, 23 alcohols, 13 alkanes, 7 ketones, 6 acids, 3 phenols, 2 aldehydes and 10 other kinds of volatile compounds) and microorganism profiles (284 and 15 genera of bacteria and fungus were detected, respectively) have been investigated during millet Huangjiu fermentation, their correlations were also established and analyzed. *Bacillus* was principal followed by *Paenibacillus* and *Weissella* form the genus level. Overall, 537 correlations were established between flavor compounds and microbes during millet Huangjiu fermentation. 153 microorganisms were relevant to the formation of main flavor compounds (*P<0*.*05*). The top five dominant genus of flavor producing microbes were *Chryseobacterium*, *Sporolactobacillus*, *Psychrobacter*, *Sphingobium* and *Anoxybacillus*. Our research provides essential information on the relationship between microbial community and the flavor formation during millet Huangjiu fermentation, and is beneficial for the development of Huangjiu products.

## Supporting information

S1 Table Dynamic change of organic acids found in Huangjiu brewed from millet during fermentation. (XLSX)

S2 Table Dynamic change of volatile compounds in Huangjiu brewed from millet during fermentation. (XLSX)

S1 Fig Distributions of α diversity indices (A: observed species index, B: Shannon diversity index) during fermentation stages
(TIF)
