# Supplementary figures and images for: The changes of microbial diversity and flavor compounds during the fermentation of millet Huangjiu, a traditional Chinese beverage
Source: PLoS One. 2022 Jan 5;17(1):e0262353. doi: 10.1371/journal.pone.0262353 (PMC8730391; doi:10.1371/journal.pone.0262353)

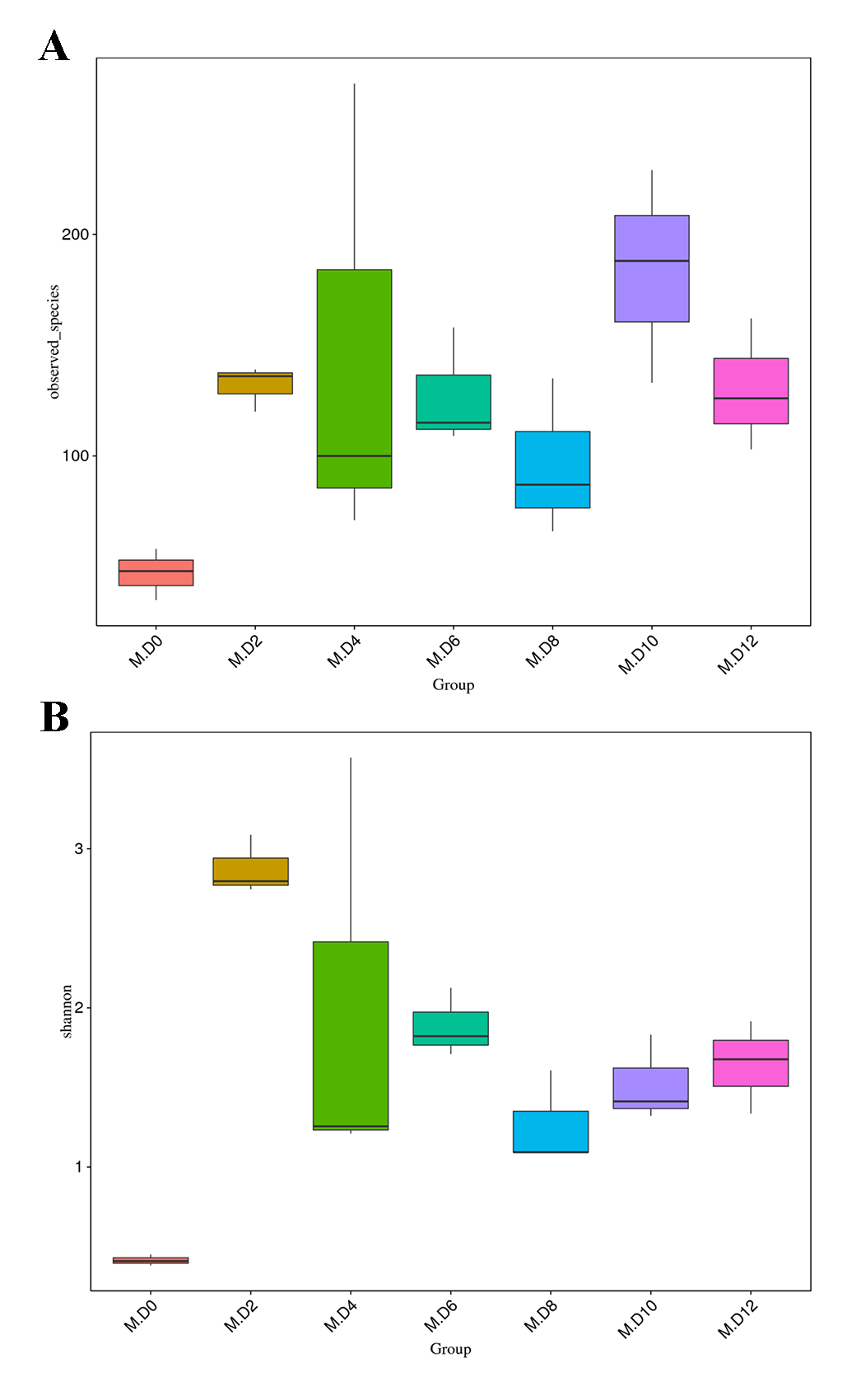

Supplement: S1 Fig — Distributions of α diversity indices (A: observed species index, B: Shannon diversity index) during fermentation stages (TIF) [file pone.0262353.s003.tif]
